# Nonequilibrium band occupation and optical response of gold after ultrafast XUV excitation

**DOI:** 10.1038/s41598-022-08338-2

**Published:** 2022-03-18

**Authors:** Pascal D. Ndione, Sebastian T. Weber, Dirk O. Gericke, Baerbel Rethfeld

**Affiliations:** 1 Department of Physics and OPTIMAS Research Center, Technische Universität Kaiserslautern, Erwin-Schrödinger-Straße 46, 67663 Kaiserslautern, Germany; 2 Department of Physics, Centre for Fusion, Space and Astrophysics, University of Warwick, Coventry, CV4 7AL UK

**Keywords:** Condensed-matter physics, Electronic properties and materials, Physics, Plasma physics, Laser-produced plasmas

## Abstract

Free electron lasers offer unique properties to study matter in states far from equilibrium as they combine short pulses with a large range of photon energies. In particular, the possibility to excite core states drives new relaxation pathways that, in turn, also change the properties of the optically and chemically active electrons. Here, we present a theoretical model for the dynamics of the nonequilibrium occupation of the different energy bands in solid gold driven by exciting deep core states. The resulting optical response is in excellent agreement with recent measurements and, combined with our model, provides a quantitative benchmark for the description of electron–phonon coupling in strongly driven gold. Focusing on sub-picosecond time scales, we find essential differences between the dynamics induced by XUV and visible light.

## Introduction

The relaxation behaviour is an essential ingredient when modelling the interaction of short-pulse lasers with matter, in particular for strong excitations, when matter is eventually pushed into a different phase^1–4^. Extreme conditions, as they occur in planets^5,6^, stars^7^ and also inertial confinement fusion^8^, can indeed only be created and probed as a transient state in the laboratory. Theoretical models are needed here in two different ways: they provide estimates for the properties of highly excited matter that is created, and they also support the diagnostics by linking the microscopic states to the global behaviour measured. Interpreting transmission or reflectivity data^9–12^, X-ray or electron diffraction patterns^13–15^, or spectra from X-ray Thomson scattering^16–18^ and X-ray near-edge spectroscopy^19,20^ are examples where such models are required, in particular, for nonequilibrium conditions.

Qualitatively, the pathway towards an equilibrium state follows a number of more or less well-separated stages^21,22^: first the electrons in each band equilibrate to respective Fermi distributions allowing the use of a temperature for later times. Holes in deep core states are filled on the same time scale. As energy transfer is more efficient than particle transfer between different bands, a common electron temperature is first established and then the densities of the different bands equilibrate with respect to the new elevated temperature^23,24^. Finally, the electron and lattice/ion temperatures equilibrate. Both lattice heating and strong changes in the electronic structure can trigger phase transitions^13,14,25^. Despite the consensus on the general behaviour, the time scales of the different stages and the importance of specific processes are highly debated^4,26–29^.

Advances in short-pulse lasers and, especially, free electron lasers (FELs)^30–33^ allow for a new class of precision experiments that challenges theoretical modelling for both the relaxation pathways and the behaviour of the nonequilibrium states created. Moreover, FELs can provide radiation in the XUV and X-ray regimes that predominantly couples to core states and, thus, initiate a different excitation and relaxation pathway than visible light. Up to now, experiments employ pulse lengths that can resolve all relaxation stages mentioned above, except the fully kinetic stage, *i.e.*, before the electrons have established a Fermi distribution^4,12^. Thus, a comprehensive theoretical model that allows a one-to-one comparison with the experiment needs to include the upper bands of the target material, at least one core state and all processes that are driven by the new, elevated electron temperature.

In this contribution, we construct a model that meets the challenges mentioned above taking gold as an example as it exhibits a non-trivial band structure and existing experimental results can serve as a benchmark. To match the experimentally accessible time scale, we exclude the fully kinetic stage and consider Fermi distributions with a well-defined temperature in all bands but trace all relaxation stages that follow. As an example for a core state, we couple the 4*f*-state to the dynamics of the 5*d*- and 6*sp*-bands.

The results of this model are compared to experimental data which yields an excellent agreement. In particular, the fast conductivity drop caused by the strongly changing occupation of the *sp*-band in the sub-picosecond range is matched well. Moreover, the experimental data for delays of several picoseconds allow for benchmarking the energy transfer between the electron and the phonon systems. Comparing these data with our results clearly rules out the most commonly used model for the coupling constant^34^ but gives good agreement for a model predicting much slower energy transfer^35^. Comparing excitations with XUV and visible light, we observe a qualitatively different evolution of the optically active *sp*-density which can be traced back to different relaxation pathways on sub-picosecond time scales.

## Results

### Three-band rate equations

We study the relaxation behaviour in solid gold excited by short XUV and X-ray pulses as provided by FELs. Such radiation can promote electrons from deeper levels into the conduction band which requires to consider the dynamics between, at least, three optically active bands or levels. As an example, we use here the 4*f*-state in addition to the 5*d*- and 6*sp*-bands that always need to be considered as there is no band gap between the *d*-electrons and the delocalised *sp*-electrons in gold.

The fully kinetic stage of the relaxation process lasts only a few femtoseconds as has been shown by solving kinetic equations^29,36^ and time-dependent DFT simulations^37^. Afterwards, a Fermi distribution has been established in each band. Here, we assume that the distributions in the *d*- and *sp*-bands have a common temperature but still nonequilibrium occupations, that is, different chemical potentials. Accordingly, the occupation of each band and the total energy content of the electrons are required to describe the system dynamics. For times beyond a picosecond, the energy transfer from the electrons to the lattice must be included as well.

The dynamics in the electron system is initiated by the photo-excitation of a core level, the 4*f*-level in our case. These electrons increase the density of the *sp*-band and the excess energy heats the electron system. The holes in the 4*f*-states are filled by *d*-electrons within 2.27 fs^38^. This process has a large impact on the occupation and energy density of the *d*-electrons as well as the *sp*-electrons: one *d*-electron fills the hole while in an Auger-like process another *d*-electron is promoted into the conduction band, the *sp*-band. Both, the initial photo-electrons as well as the Auger electrons can be excited far above the Fermi energy into quasi-free states of the *sp*-band. Both processes also release a large amount of kinetic energy into the electron system. After photo- and Auger-excitation, the occupations of the *d*- and *sp*-bands are not in equilibrium with the new energy content, *i.e.*, the temperature. The time of the subsequent equilibration process between the two bands is quantified here by a relaxation time that describes the imbalance of the rate of impact ionisation of *d*-electrons by hot *sp*-electrons and the rate of deexcitation of *sp*-electrons into the *d*-band.

The processes described above are cast into a system of rate equations that can also be easily extended to include more states. The energy in the electron system is traced by enforcing energy conservation in each process. Moreover, we couple the electrons to the phonons via a well-established two-temperature model^39^ with the electron–phonon energy transfer rate $g_{ei}$ as the critical parameter. Thus, our prediction for the evolution of the band occupation spans the large range of relaxation stages after the fully kinetic phase of a few femtoseconds to the equilibration of electron and phonon temperatures. Including all processes and boundary conditions mentioned above, ﻿we obtain

1a $$\begin{aligned} \frac{d n_{sp}}{d t}&= \frac{S}{\hbar \omega _{L}} + \frac{n_{d}}{\tau _{\mathrm{Auger}}}\left( 1-\dfrac{n_{f}}{n_{f}^\mathrm{ini}}\right) -\dfrac{1}{\tau _{\mathrm{relax}}} \left( n_{sp} - n_{sp}^{eq}\right) \,, \end{aligned}$$

1b $$\begin{aligned} \frac{d n_{d}}{d t}&=\,\qquad -2 \frac{n_{d}}{\tau _\mathrm{Auger}}\left( 1-\frac{n_{f}}{n_{f}^{\mathrm{ini}}}\right) +\frac{1}{\tau _{\mathrm{relax}}} \left( n_{sp} - n_{sp}^{eq}\right) \,, \end{aligned}$$

1c $$\begin{aligned} \dfrac{d n_{f}}{d t}&= -\dfrac{S}{\hbar \omega _{L}} + \dfrac{n_{d}}{\tau _{\mathrm{Auger}}}\left( 1-\frac{n_{f}}{n_{f}^\mathrm{ini}}\right) \,, \end{aligned}$$

1d $$\begin{aligned} C_{e} \, \frac{dT_{e}}{dt}&=S- \Delta E_f^{sp}\, \frac{S}{\hbar \omega _{L}} + \Delta E_f^{d} \, \frac{n_{d}}{\tau _\mathrm{Auger}}\left( 1-\dfrac{n_{f}}{n_{f}^\mathrm{ini}}\right) -g_{ei}\left( T_{e}-T_{i}\right) \,, \end{aligned}$$

1e $$\begin{aligned} C_{i} \, \frac{dT_{i}}{dt}&= g_{ei} \left( T_{e}-T_{i}\right) \,. \end{aligned}$$

Here $n_a$ labels the electron density in the *a*-band and $T_e$ and $T_i$ are the electron and lattice (ion) temperatures, respectively. $S = S(t)$ describes the original energy source, that is, the energy input from photons with frequency $\omega _L$. Thus, $S / \hbar \omega _L$ is the creation rate of direct photo-electrons. $\tau _{\mathrm{Auger}}$ is the Auger time for the deep level and $\tau _{\mathrm{relax}}$ is the time constant for the equilibration between the densities in the *d*- and *sp*-bands. The details of the band structure, which has been calculated from DFT simulations^40^, enter the model via the equilibrium densities at elevated temperatures $n_a^{eq}$ and the electron heat capacity $C_e$. $n_f^{\mathrm{ini}}$ labels the density of the 4*f*-state before excitation. $\Delta E_f^{sp}$ is the energy difference from the 4*f*-state to the Fermi energy in the *sp*-band and $\Delta E_f^{d}$ is the energy gap from the 4*f*-state to the *d*-edge.

Except for two parameters, the relaxation time between the *d*- and *sp*-bands and the electron–phonon coupling, all input parameters are measured quantities or can be generated by *ab initio* simulations. In turn, models for the important parameters $\tau _\mathrm{relax}$ and $g_{ei}$ can be benchmarked by a quantitative comparison of model predictions and experimental data for the electron dynamics as we will demonstrate on the example of electron–phonon energy equilibration.

### Density and temperature relaxation

We demonstrate the capabilities of the three-band model (1) by a direct comparison with a recent experiment^12^. To that end, we consider excitation of gold with an XUV pump pulse of ${150}\,{\hbox {fs}}$ duration (full width at half maximum), a wavelength of ${13.6}\,{\hbox {nm}}$ and a total excitation energy of 0.89 ${\hbox {MJkg}}^{-1}$. For this photon energy, the photo-electrons are excited 5.45 eV above the Fermi energy while the Auger electrons will be ${>\!\!70}\,{\hbox {eV}}$ more energetic. For the model parameters, we choose a density relaxation time of $\tau _{\mathrm{relax}} = {200}\,{\hbox {fs}}$ (see “Methods” section) and two models for the electron–phonon coupling parameter $g_{ei}$ according to Refs.^34,35^, respectively.

**Figure 1:**
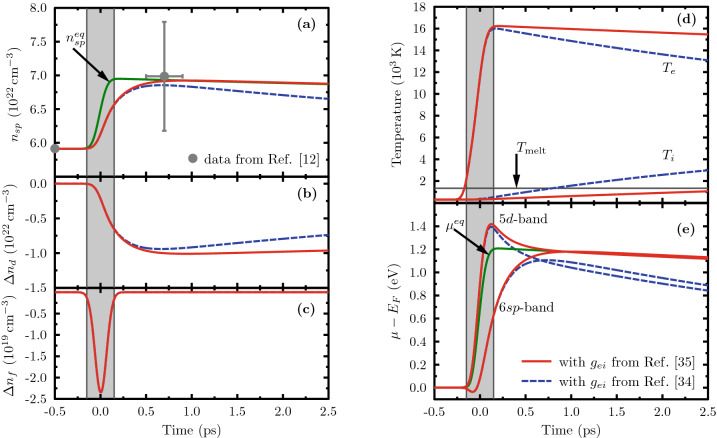
Electron response to XUV excitation of gold: (**a**) evolution of the 6*sp*-band density; (**b**) response of the 5*d*-band density; (**c**) direct excitation of the 4*f*-shell; (**d**) evolution of the electron and ion temperatures; (**e**) the transient chemical potentials relative to the Fermi energy $E_F$ for the 6*sp*- and 5*d*-electrons. The grey shaded area represents the time when the pump pulse is active. The full red lines and the blue dashed lines follow from calculations with the electron–phonon coupling according to Ref.^34^ and Ref.^35^, respectively. The green line assumes instant equilibration of the 5*d*- and 6*sp*-bands. The experimental data point in panel (**a**) is taken from Ref.^12^.

Figure 1a–c show the temporal evolution of the electron density in the three active bands, *i.e.*, the 6*sp*- and 5*d*-bands as well as the 4*f*-core state. The external excitation partially depopulates the 4*f*-state, but these core holes are quickly filled again by Auger processes. With an Auger time of ${2.27}\,{\hbox {fs}}$, the population of the 4*f*-state roughly follows the laser intensity.

The direct changes in the *sp*- and *d*-band densities due to laser excitation and Auger recombination are pretty small. Much more important is the large amount of energy each process brings into the *sp*-band that induces a strong nonequilibrium between its energy content, thus temperature, and its occupation. This imbalance triggers very effective impact ionisation of *d*-electrons that dominate the changes visible in Fig. 1a,b until a quasi-equilibrium is reached after roughly ${700}\,{\hbox {fs}}$. For longer times, electron–phonon coupling cools the electrons and the *sp*-density decreases accordingly. To quantify the effect of the relaxation between the *sp*- and *d*-bands, we included the result assuming instant equilibration between these bands. Although the data point from Ref.^12^ is matched well, it cannot be used to distinguish between the models due to the large error bars in this inferred quantity.

The electron temperature, describing the energy content of the upper bands, is shown in Fig. 1d. Both direct laser excitation and Auger processes strongly drive it during the laser pulse. Then, it decays on a picosecond time scale due to electron–phonon coupling. The increasing lattice temperature exceeds melting temperature long before it equilibrates with the electron temperature, again in rough agreement with the experimental data of Ref.^12^. Note that the material can be in the solid phase after the melting temperature has been reached as thermal melting requires considerable time^4,41^.

The nature of the nonequilibrium created can be captured best when focusing on the chemical potentials of the *sp*- and *d*-bands as shown in Fig. 1e. The nonequilibrium band occupation is evident by the strong differences between the two chemical potentials during and shortly after the excitation. Similar to the densities, the chemical potentials equilibrate on a time scale of ${700}\,{\hbox {fs}}$. However, the energy loss to the phonons slightly drives the band occupation out of equilibrium, persistent even on the long time scale of electron–phonon equilibration. Such behaviour has also been observed for the very different case of ultrafast magnetisation of itinerant ferromagnets^42^. It proves that signatures of electronic nonequilibrium can be present on much longer time scales than suggested by the relaxation time^29,43^. Of course, this effect is more prominent for the case with stronger electron–phonon coupling.

### Time-resolved DC electrical conductivity and scattering frequencies

The optical properties, that can be directly observed in experiments, provide a much better test for the quality of our model than the *sp*-density. Ref.^12^ provides data for the DC electrical conductivity. Within the Drude model, it can be expressed by the conduction band density $n_{sp}$ and a density- and temperature-dependent collision frequency $\nu _{\mathrm{tot}}$ as

2 $$\begin{aligned} \sigma _{0}\left( t \right) = \frac{e^2 \; n_{sp}(t)}{ m_{sp} \; \nu _{\mathrm{tot}}(t)}\,, \end{aligned}$$

where *e* is the electron charge and $m_{sp}$ the effective electron mass in the *sp*-band.

For the complex band structure in gold, the scattering rate $\nu _{\mathrm{tot}}$ has two components: scattering of conducting *sp*-electrons with phonons and with *d*-band electrons. We apply the low-temperature form of electron–phonon scattering and take $\nu _{ei}$ to increase linearly with the phonon (ion) temperature $T_i$. For the electron–electron scattering rate $\nu _{ee}$, we follow Fourment et al.^44^ and take it to be proportional to the densities of both electrons and holes in the *d*-band. Thus, we have

3 $$\begin{aligned} \nu _{\mathrm{tot}}(t)&= \nu _{ee}(t) + \nu _{ei}(t) \nonumber \\ &= A \, n_d (t) \left[ n_d^{\mathrm{full}} - n_d (t) \right] + B \, T_i(t) \,, \end{aligned}$$

where $n_d^{\mathrm{full}}$ describes a full *d*-band and the coefficients *A* and *B* have been determined by experimental data (see “Methods” section). The time-dependent temperature and density are provided by our relaxation model (1) which, through Eqs. (2) and (3), also determine the conductivity of the evolving system.

**Figure 2:**
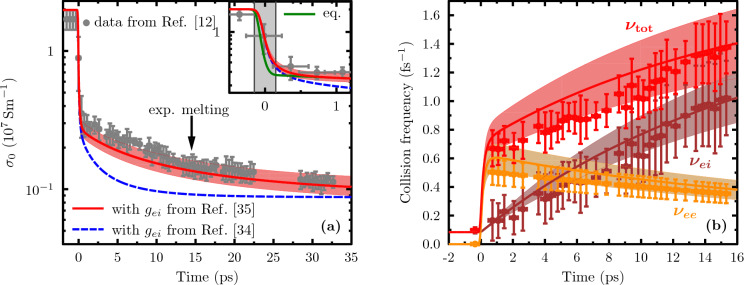
Transient DC conductivity and scattering frequencies in XUV excited gold. Results of our model (lines) are compared to experimental data (symbols with errorbars) taken from Ref.^12^ where the shaded areas reflect the uncertainty of the absorbed energy in the experiment. The modelling uses time-dependent band occupations and temperatures from the three-band rate equations (1) as shown in Fig. 1. (**a**) DC conductivity with electron–phonon coupling applied according to Ref.^35^ (red solid line) and Ref.^34^ (blue dashed line). Note the logarithmic scale of the conductivity $\sigma _0\left( t\right)$ when assessing the deviations. The arrow indicates the time of melting as observed in the experiment. The inset compares our full modelling with data assuming instant equilibration between the bands (green line). (**b**) Total electron scattering rate (red) and its contributions from electron–phonon scattering (brown) and electron–electron scattering (orange).

In Fig. 2, we compare the predictions for the evolution of the DC conductivity $\sigma _0$ and the scattering rates of excited gold with experimental data from Ref.^12^. The shaded area accounts for the uncertainty of the absorbed energy in the experiment around a mean value of ${0.89}\,\hbox {MJkg}^{-1}$ represented by the line. Figure 2a shows an abrupt and fast decrease of the DC conductivity directly during the XUV laser pulse, followed by a more shallow decay on picosecond time scales. The strong conductivity drop is mainly caused by a strong increase of the scattering rate $\nu _{\mathrm{tot}}$ shown in Fig. 2b together with the contributing frequencies $\nu _{ee}$ and $\nu _{ei}$. In cold gold, the rates are very small since Pauli blocking prevents scattering. The XUV pulse creates holes in the *d*-band by Auger recombination of the 4*f*-state and, in particular, further impact ionisation by energetic *sp*-electrons and is thus opening an efficient scattering channel. This newly opened way of scattering leads to a strong increase of $\nu _{ee}$ already during the excitation process.

On a picosecond time scale, energy is transferred to the phonons (ions) leading to an almost linear rise of $\nu _{ei}$ with time. The increase of $\nu _{\mathrm{tot}}$ is more shallow due to a slight decrease of $\nu _{ee}$ in this phase. Together with a decreasing *sp*-density, this behaviour leads to a weak decay of the DC conductivity, *cf.* Fig. 2a. We have employed two different models for the electron–phonon coupling: the often-used model of Lin et al.^34^ and the model of Medvedev & Milov^35^ which predicts much slower energy transfer. We find that the experimental data^12^ agree over the complete time scale very well with the theoretical curve using the coupling strength of Ref.^35^ for both the conductivity and the scattering rates. In contrast, the Lin model^34^ neither reproduces the qualitative nor the quantitative behaviour of the conductivity (note the logarithmic scale in the figure). It decreases too strongly and saturates too early, resulting from a too fast and too strong increase of the electron–phonon scattering rate $\nu _{ei}$. In contrast to the *sp*-density, the error bars for the more directly obtained conductivity are small enough to clearly distinguish between these energy transfer models. This comparison demonstrates the strength of our model to provide an important benchmark for the controversially discussed electron–phonon coupling parameter^34,35,45,46^. The model presented here was developed for a material with a stable lattice and melting occurs within the time scale shown. In the experiment, a loss of lattice stability was found after ${14.5}\,{\hbox {ps}}$ for the excitation conditions used here^12^. Although our model is not strictly applicable, the DC conductivity calculated compares well with the experimental data far beyond this time. This interesting fact suggests that the collision rates and conduction band density do not change considerably during the phase transition.

The inset of Fig. 2a highlights the requirements if the second parameter of our model, $\tau _{\mathrm{relax}}$, should be determined by experiments as well. For that end, we also have included calculations assuming instant relaxation, that is, both bands share a temperature and chemical potential. Unfortunately, present experimental data do not have the time resolution and data quality to distinguish these curves. However, dedicated experiments should be, in principle, able to resolve the sub-picosecond relaxation with the pulse structure of existing FELs^47,48^.

### XUV versus optical excitation

We find strong qualitative differences in the relaxation behaviour after ultrafast excitation with an optical laser pulse as compared to the XUV excitation discussed above. In the XUV case, the entire laser energy is absorbed within the 4*f*-core shell. The Auger process creates highly energetic electrons and, accordingly, a high temperature in the conduction band. As a result, further ionisation of the *d*-band occurs when the chemical potentials equilibrate (*cf.* Fig. 1). In contrast, visible light is absorbed by *d*- and *sp*-electrons and a reduced system of rate equations can be set up for this case^49^. At ${400}\,{\hbox {nm}}$ laser wavelength, most of the energy is absorbed by 5*d*-electrons, exciting them to free states of the conduction band at energies slightly above Fermi level. In this case, much more electrons are excited than in the XUV case if the same total energy is absorbed. Although we have the same relaxation term as in Eqs. (1a) and (1b), after optical excitation the relaxation drives electrons back into the *d*-band^49^, even before the coupling to the phonons further cools the conduction electrons.

**Figure 3:**
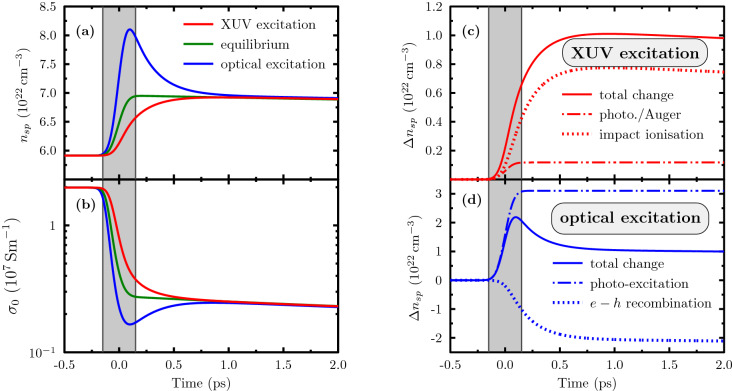
Comparison of excitations with XUV and optical pulses. (**a**) Density response and (**b**) evolution of the DC conductivity of gold after absorption of ${13.6}\,{\hbox {nm}}$ (red lines) and ${400}\,{\hbox {nm}}$ (blue lines) photons during the time indicated by the shaded area. The green line assuming instant equilibration is independent of the excitation process. All modelling considers electron–phonon coupling according to Ref.^35^. (**c**) Total density change of the 6*sp*-band (solid line) due to an excitation with XUV photons, together with the individual contributions due to direct photo-excitation and Auger filling of 4*f*-holes (dashed dotted line - both processes yield identical contributions) and impact ionisation of *d*-electrons (dots). (**d**) Total density variation in the 6*sp*-band (solid line) for optical excitation and its contributions via direct photo-absorption (dashed dotted line) and the losses due to filling of holes in the *d*-band (dots).

Figure 3a,b quantify the differences of optical and XUV excitation as described above for the electron density in the 6*sp*-band and the DC conductivity. For both excitations, the absorbed energy is the same and, thus, both systems relax to the same equilibrium. However, the intermediate phase is very different: we observe an underpopulation of the *sp*-band (compared to the quasi-equilibrium state determined by the electron temperature at this time) after XUV excitation, whereas the optical laser strongly overpopulates the conduction band initially, *i.e.*, it creates a density above the quasi-equilibrium value. In the latter case, the very strong increase of density is followed by a slightly longer relaxation to its equilibrium value. These differences are also reflected in the DC conductivity which is mainly determined by the conduction band density and the free states in the *d*-band.

To further elucidate the origin of the different relaxation behaviour, we investigate the different excitation channels and relaxation terms in our rate equations in more detail. In Fig. 3c,d, we plot the integrated density-change of the 6*sp*-band for different processes separately. For XUV photons, the initial photo-ionisation, as well as the Auger processes, give very small contributions. They are equal in magnitude and their integrated contribution remains constant after the pulse or Auger decay time, respectively. However, these processes created few highly excited electrons that efficiently trigger collisional excitation promoting many more *d*-electrons to the *sp*-band. Thus, this process is the main contributor in the relaxation phase and even throughout the XUV pulse. In contrast, photo-excitation of *d*-electrons is the only, but very efficient, process driving the *sp*-density increase for optical excitation (see Fig. 3d). Since the resulting density exceeds its equilibrium value by far, the subsequent relaxation is mainly driven by the recombination into *d*-band holes. Of course, the observable deviation from the equilibrium density is strongly influenced by the relaxation time $\tau _{\mathrm{relax}}$ which is here a free parameter. Recent data^50^ showing strong recombination into the *d*-band already during the laser pulse thus indicate a shorter relaxation time than used here.

## Conclusion

In summary, we have studied the dynamics of band occupation and DC conductivity in gold excited by ultrashort pulses of high-energy photons. For that goal, we have developed a system of rate equations that describes the evolution of electron density in the *sp*- and *d*-bands as well as a core state. Our model also tracks the energy content in the upper bands and couples this energy to the phonon systems. The results were employed to calculate the dynamics of the DC conductivity which allows for a more direct comparison to experiments. This comparison yields excellent agreement for both the sub-picosecond dynamics governed by the equilibration of electron densities within the *sp*- and *d*-bands and the evolution on several tens of picoseconds driven by the electron–phonon energy transfer. The latter allows us to clearly rule out an often-applied model for the electron–phonon coupling parameter. This fact demonstrates the strength of our model to bridge between theoretical estimates of important transport and relaxation parameters, which are usually done for static conditions, and highly dynamic experiments. This tool is particularly important for states with high energy density that prohibit static conditions in the laboratory. We also elucidate the different relaxation pathways for excitations with XUV and visible light: whilst secondary impact ionisation is the dominant process for XUV irradiation, direct photo-excitation prevails for optical lasers. These insights highlight the complex physical processes driving the transient electronic occupation and material response at a microscopic level that need to be accounted for when predicting and interpreting experiments with short-pulse excitations. Our model is also directly applicable to other noble metals, *e.g.*, copper and silver. However, an application of the rate equations for transition metals will require adjustments, in particular, to the description of the excitation process. Combined with experiments having improved time resolution, the modelling of the occupational nonequilibrium can thus establish the time scales and pathways of the relaxation processes after ultrafast excitations.

## Methods

### The three-band model

The density response is described by the rate equations (1) with the assumption of a Fermi distribution in each band. In the XUV case, we consider heating by a pulse of photons with a wavelength of ${13.6}\,{\hbox {nm}}$ (${91.2}\,{\hbox {eV}}$) that inject ${0.89}\,\hbox {MJkg}^{-1}$ of energy in solid gold. We consider a Gaussian pulse form with a full width at half maximum of ${150}\,{\hbox {fs}}$. Due to their high energy, each photon excites one electron from the 4*f*-state directly into the *sp*-band.

**Figure 4:**
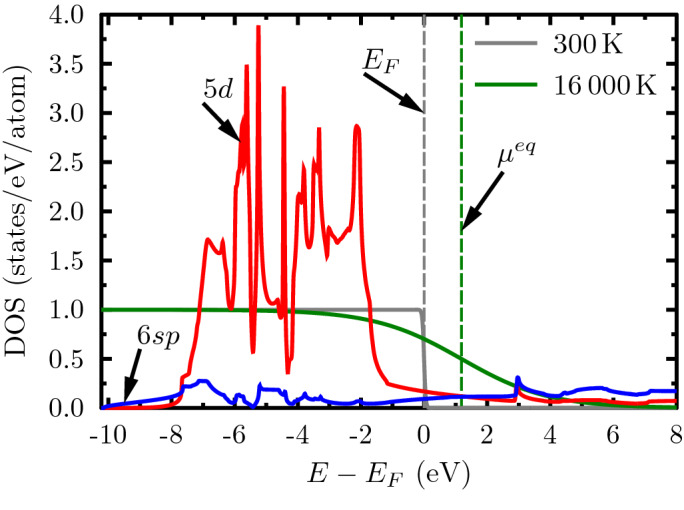
Partial density of states for the 5*d*- and 6*sp*-bands of gold. The equilibrium Fermi distributions for 300 K and 16,000 K are shown and the shift in chemical potential after heating is indicated. The energy scale is given relative to the Fermi energy.

The energy landscape for the electrons is given by the band-resolved density of state which have been calculated by density functional theory^40^. Prior to the excitation, the electron configuration of gold is $[Xe]4f^{14}5d^{10}6s^{1}$ corresponding to a fully occupied *d*-band and one electron in the conduction band. Although, we consider the density of state to be unchanged by the relatively small excitation, the equilibrium chemical potential will shift due to the increased temperature. Both the partial density of states and the temperature effects on the Fermi functions and chemical potential are shown in Fig. 4. The density of states was calculated for the ground state by the all-electron full-potential linearised augmented-plane wave code *Elk* using an $80\times 80\times 80$ ***k***-point grid, spin-orbit coupling and the local density approximation^40^. We consider the DOS to remain unchanged from the ground state which has been shown to be a good approximation for the range of electron temperatures used here^51^.

The set of coupled rate equations (1) requires a number of material constants. The rate of direct excitation of core electrons into the conduction band is given by the absorbed energy and the photon energy. The core holes in the 4*f*-states considered here are filled rapidly by *d*-electrons via Auger processes with decay times of ${2.35}\,{\hbox {fs}}$ and ${2.19}\,{\hbox {fs}}$ for the $4f_{5/2}$ and $4f_{7/2}$ states, respectively^38^. For practical reasons, we use the average of these times.

After the initial excitation and Auger recombination, we have an imbalance of electron occupation in the *sp*- and *d*-bands. The balance of recombination into free *d*-states and further impact ionisation into the *sp*-band will establish equilibrium densities that can be determined by

4 $$\begin{aligned} n_{a}^{eq} = \int \mathrm {d}E \, f\left( E, \mu ^{eq}, T_e\right) D_{a}\left( E\right) , \end{aligned}$$

where *a* labels the band, $D_{a}$ is the partial density of states, $f\left( E, \mu ^{eq}, T_e\right)$ is the common Fermi distribution for both bands with the chemical potential $\mu ^{eq}$ being obtained by conservation of total number of electrons^52^. The relaxation of the band occupations towards this equilibrium density is described by the time scale $\tau _{\mathrm{relax}}$ and, without any other processes, we would observe an exponential decay of the imbalance in the *d*- and *sp*-band densities on this time scale. Here, we have chosen $\tau _{\mathrm{relax}} = {200}\,\hbox {fs}$. This time scale is within existing bounds: it should be considerably longer than the fully kinetic phase that establishes Fermi-like distributions in each band and takes a few femtoseconds in the given energy range^29,36,37^ and it must be shorter than the time resolution of previous measurements (${540}\,{\hbox {fs}}$) that could be described by equilibrium densities^9,53^. The value we have taken here is thus near the upper limit to yield estimates for the minimum time resolution an experiment must have to investigate these effects.

The energy balance between the electrons and phonons is described within the two-temperature model^39^. The ion heat capacity is taken to be constant $C_i = {2.327}\,\hbox {MJm}^{-3}\hbox {K}^{-1}$ (see Ref.^54^). In contrast, the electron heat capacity $C_e$ is strongly temperature-dependent and it is calculated as the temperature derivative of the internal energy at each time step

5 $$\begin{aligned} C_e(T_e) = \frac{\partial }{\partial T_e} \int \mathrm {d}E \, E \; f(E, \mu ^{eq}, T_e) \; \big [D_{sp}(E)+D_d(E)\big ]\,, \end{aligned}$$

where the total density of states is expressed by the partial density of states of *d*- and *sp*-electrons, $D_{sp}$ and $D_d$, respectively.

For the binding energies of the 4*f*-states used in the three-band-model (1d), we apply the average value of the $4f_{5/2}$ and $4f_{7/2}$ doublet with energies relative to the Fermi energy of ${87.6}\,\hbox {eV}$ and ${83.9}\,\hbox {eV}$ , respectively^55,56^.

The integration of the rate equations (1), that represent coupled nonlinear ordinary differential equations, has been performed by the Runge-Kutta scheme of order five. We also tested an implicit scheme with the Adams-Bashforth-Moulton multistep method of order five which yielded the same results for the integrated quantities.

### Optical excitation

To describe gold excited by an optical laser pulse with ${400}\,{\hbox {nm}}$ (${3.1}\,\hbox {eV}$), we must only consider two optically active bands, namely the 5*d*- and 6*sp*-electrons as shown in Fig. 4. Moreover, the energy can be absorbed by both the 5*d*- and the 6*sp*-electrons. For such system, we have developed a dynamic two-band model^49^ similar to Eqs. (1) that account for photo-absorption, excitation of *d*-electrons and deexcitation of *sp*-electrons as well as the energy transfer to the phonons. The ratio of intra- and interband photo-absorption is set to be proportional to the number of electrons available, as well as to the number of free states for these electrons after excitation in order to account for Pauli blocking. As in the three-band model, the time scale for the equilibration of the band occupation is included as a free parameter.

### Band-resolved chemical potentials

The dynamics of the chemical potentials shown in Fig. 1e directly follows from the time-dependent band occupation. For each band *a*, we have

6 $$\begin{aligned} \frac{d \mu _a}{dt} = \frac{1}{P^\mu _a} \left( \frac{d n_a}{d t} - P^T_a \frac{dT_e}{dt} \right) \,. \end{aligned}$$

Here, the nonlinear parameters $P^\mu _a$ and $P^T_a$ are evaluated at each time step via

7a $$\begin{aligned} P^\mu _a(t)&= \frac{\partial n_a}{\partial \mu _a} = \int \mathrm {d} E \; D_a(E) \frac{\partial f_a(E, \mu _a(t), T_e(t))}{\partial \mu _a} \,, \end{aligned}$$

7b $$\begin{aligned} P^T_a(t)&= \frac{\partial n_a}{\partial T_e} = \int \mathrm {d} E \; D_a(E) \frac{\partial f_a(E, \mu _a(t), T_e(t))}{\partial T_e} \,. \end{aligned}$$

### DC conductivity and scattering rates

The Drude model for the electrical conductivity (2) and the scattering rates (3) require a number of parameters that have been determined from experimental data. For the effective electron mass of the *sp*-band, we use the free-electron mass^57^. The constant *B* for the electron–phonon scattering is determined via its cold value $B = \nu _{ei}^\mathrm{cold}/{300}\,{\hbox {K}}$, where $\nu _{ei}^\mathrm{cold}={0.084}\,{\hbox {fs}^{-1}}$ is taken from data of Refs.^57,58^. The parameter *A* for the electron–electron scattering (3) is found to be almost constant at elevated electron temperature and set to be $A= V^2 \cdot {0.36}\,{\hbox {fs}^{-1}}$^44^ with the volume of the unit cell $V={1.69\times10^{-29}}\,{\hbox {m}^3}$.

## Data availability

The data supporting the findings of this study are available from the corresponding author upon reasonable request.
